# Supplementary material for: Structural limits of single-barrier reform in algorithmic recourse: A formal series-system model with implications for digital health
Source: PLOS Digit Health. 2026 Oct 26;5(10):e0001784. doi: 10.1371/journal.pdig.0001784 (PMC13705585; doi:10.1371/journal.pdig.0001784)
Supplement: Supplementary file 1 [file pdig.0001784.s002.pdf]

Overall impression

This is an ambitious and intellectually interesting manuscript. Its core argument is clear: when recourse barriers are arranged as a multiplicative, series-structured system, single-barrier reforms will have very small absolute effects, whereas coordinated multi-layer reform can matter materially. The paper is well organised, the research question is explicit, the mathematical propositions are easy to follow, and the manuscript is commendably transparent in disclosing several limitations, including that the simulations are not independent validation of the propositions and that the robustness claims apply only within author-defined perturbation ranges. The supplementary file is useful and broadly consistent with the main text, including the headline findings of baseline success 0.0018%, maximum single-barrier gain 0.0055%, and dominant three-way interaction.

However, the manuscript is not ready for acceptance in its current form. My main concern is that the strongest conclusions are driven primarily by the model structure rather than by healthcare-specific empirical evidence. The manuscript explicitly states that it is a formal abstraction, that barrier probabilities are mapped from heterogeneous sources, and that the principal conclusions are analytical properties of the series structure. That is very valuable, but it also means the paper should be positioned much more carefully. At present, the discussion sometimes reads as though the work has demonstrated how healthcare recourse actually behaves, whereas the paper more securely shows what follows if recourse is represented as an 11-stage multiplicative series system parameterised largely from cross-domain proxy data.

My overall recommendation is **major revision**. The paper has potential, but the framing, claims, and empirical grounding need to be tightened substantially before publication.

Fit for PLOS Digital Health

The topic is relevant to PLOS Digital Health in a broad sense. The manuscript addresses clinical AI governance, fairness, recourse, and sociotechnical barriers, all of which are compatible with the journal's scope. The introduction and discussion explicitly position healthcare AI as the primary context and connect the work to clinical machine learning fairness and health dataset bias.

That said, the fit is only **partial in the current version**. The paper repeatedly states that healthcare is the primary context, yet most of the empirical parameterisation comes from CFPB, FTC, and Legal Services Corporation sources, with only one barrier parameter directly linked to a healthcare algorithmic audit. The manuscript itself says that healthcare is the primary context while credit, employment, and housing are used as supporting analogues "where empirical barrier data are available", and the barrier tables confirm that most inputs are drawn from non-healthcare domains.

For PLOS Digital Health, I think the manuscript would fit much better if the author did one of two things. Either:

1. strengthen the healthcare-specific empirical basis substantially, or
2. reposition the article more accurately as a cross-sector conceptual or mathematical framework with implications for digital health, rather than as a healthcare-centred empirical study.

As written, the manuscript risks overclaiming healthcare specificity relative to the underlying evidence base.

Major comments

1. The headline findings are largely consequences of the chosen model structure, not independent empirical discoveries

The paper proves Proposition 1 and Proposition 2 from the multiplicative series model, then shows simulations and decompositions that are consistent with those propositions. The manuscript also explicitly acknowledges that the simulations are “consistent with but not independent of” the propositions because both derive from the same multiplicative structure. This is a crucial admission. It means the reader should not interpret the bootstrap, Sobol, Morris, and strategy-removal analyses as independent empirical confirmation that real-world recourse systems are interaction-dominant; rather, they show that the assumed model behaves as expected under perturbation.

This does not invalidate the work, but it does change what the paper is. At present, the manuscript sometimes presents the 87.6% three-way interaction, the near-zero single-barrier effects, and the threshold behaviour as though they reveal an empirical property of healthcare recourse systems. More precisely, they reveal a structural property of a specific formalisation of recourse. The paper would be much stronger if it separated:

- theorem-level results that follow from the multiplicative model,
- simulation-based illustrations of those theorems,
- empirical assumptions used for parameterisation,
- and real-world claims that remain hypothetical until validated against observed recourse trajectories.

Without that separation, the manuscript overstates the evidential weight of its results.

2. The healthcare framing is not sufficiently supported by the parameter sources

This is my biggest concern regarding both validity and journal fit. The manuscript says healthcare AI is the primary context, yet the barrier table shows that many model inputs are derived from credit reporting, dispute resolution, legal access, and employment-related sources. The main empirical sources listed for the 11 barriers are CFPB 2022, FTC 2013, CFPB 2022 complaint data, LSC 2022, and Obermeyer et al. 2019. In other words, the majority of parameter values are not measured in healthcare recourse pathways.

This creates a major transportability problem. For example, it is not clear that credit-report correction propagation, annual report access, or justice-gap statistics can be mapped straightforwardly onto recourse after adverse healthcare algorithmic decisions such as triage, prior authorisation, or risk stratification. The manuscript states that mapping decisions were conservative, but conservatism does not solve domain mismatch.

The author should therefore do one of the following:

- provide a much more rigorous justification for each mapping into healthcare recourse,
- add healthcare-specific data for more than one barrier,
- or narrow the claims and title so that the paper is explicitly cross-domain rather than healthcare-led.

At present, the empirical foundation does not support the strength of the healthcare-specific conclusions.

3. The discussion over-relies on a single healthcare bias example without sufficient contextualisation

The manuscript relies heavily on Obermeyer et al. 2019 as its principal healthcare-specific example and as the empirical basis for the “Systemic Bias in Algorithms” barrier, yet does not sufficiently discuss the scope and limits of that study. The citation is highly important, but it reflects a particular algorithmic design problem in a specific context and cannot on its own support a comprehensive account of healthcare algorithmic bias across populations, settings, or mechanisms. The discussion would benefit from a more balanced treatment of whether the observed disparity reflects proxy choice,

broader institutional or policy design, or their interaction. The manuscript also cites more recent fairness and transparency literature, but does not use it to contextualise how the field has evolved since 2019 or to provide a more current account of bias mitigation efforts in healthcare AI. Without this clarification, the healthcare framing risks overgeneralising from one influential but context-specific study.

4. The sequential multiplicative independence assumption is under-justified and is likely a major driver of the result

The model defines successful recourse as the product of 11 pass probabilities and assumes a sequential series structure. The limitations note that the model assumes multiplicative independence between barriers and does not capture feedback loops, heterogeneity, or alternative correlation structures. Those are not minor caveats; they are central to the validity of the conclusions.

If barriers are correlated, partially parallel, iterative, or context-dependent, then the quantitative conclusions could change materially. Real-world recourse is often iterative rather than strictly serial: a patient may obtain help through multiple routes, institutions may revisit a decision, advocates may bypass some stages, and some barriers may not be conditionally independent. Yet the main conclusion of interaction dominance is almost built into the series formulation on the probability scale.

This does not mean the model is wrong, but it means the manuscript needs stronger justification for why this structure is the right abstraction for the problem. The manuscript should either justify much more rigorously why the serial multiplicative topology is the appropriate abstraction, or include at least one comparison with a plausible alternative structure, for example:

- correlated barriers,
- partially parallel pathways,
- repeated attempts or feedback loops,
- or layer-level rather than stage-level state transitions.

Without such comparisons, the paper shows that one stylised formulation has a certain property, but not that this property is robust across plausible representations of recourse.

5. Several interpretive claims are stronger than the supporting results

Several interpretive claims are stronger than the supporting results, and this overstatement begins already in the abstract and author summary and continues into the Discussion.

The paper states that Sobol indices confirmed interaction dominance, with total-order indices substantially exceeding first-order indices for all barriers. Yet in the supplementary table, the ST–S1 gap is very small for some barriers, especially Awareness Gap (0.112 vs 0.109) and Legal Knowledge Gap (0.111 vs 0.110). By contrast, other barriers do show somewhat larger gaps. So the general claim is directionally correct, but "substantially" is overstated for all barriers taken individually.

Similarly, the robustness narrative needs tightening. The supplementary SNR table classifies 25% noise as poor and shows coefficient of variation rising sharply with increasing noise, reaching 73.2% at 25% noise and 93.8% at 30% noise. The manuscript does acknowledge that true uncertainty may exceed the tested bounds, but the overall presentation still leans too heavily on the language of robustness. The safer interpretation is that the findings appear stable within the author's bounded perturbation scenarios, not that the model has been shown to be broadly robust under realistic uncertainty.

The discussion should therefore be moderated, especially where it moves from "this follows under the model" to "this explains why real-world policy reforms have had limited impact".

6. Policy implications are overstated relative to the evidence base

The discussion argues that fairness interventions limited to the algorithmic layer, such as bias mitigation or audit requirements, will have negligible population-level impact without simultaneous reform of other layers. This is plausible and may indeed be true in many settings. However, the manuscript does not actually evaluate real healthcare recourse programmes, audit regimes, or patient-notification interventions. It evaluates a stylised formal model parameterised largely from cross-sector proxies.

Accordingly, the manuscript can reasonably argue that **if** recourse behaves as a low-baseline multiplicative series system, then single-lever reforms will have limited absolute effect. It cannot yet firmly conclude that current healthcare audit or notification policies are ineffective in practice. Those statements should be reframed as implications or hypotheses rather than demonstrated policy facts.

7. Reproducibility is promising, but the derivation audit trail remains incomplete within the submitted materials

The manuscript provides code and data availability statements and states that full derivation logic is in the repository. That is positive. However, within the manuscript and supplementary file themselves, the derivation logic is still quite compressed. Table 3 gives brief mapping logic and ranges, but not enough detail for a reviewer to fully audit each transformation from source statistic to pass probability without consulting the external repository.

For a paper whose conclusions depend so strongly on parameter choice and structure, more of that derivation logic should be brought into the submitted materials themselves, either in a more detailed supplement or in an appendix. In particular, the steps from source statistics to each pass probability should be reproducible from the manuscript package alone.

Minor comments

1. The manuscript should define “successful recourse” more explicitly. Is success correction of the underlying data, reversal of the adverse decision, access to an appeal, or full resolution across all institutions? The current wording suggests full end-to-end resolution, but this should be stated operationally.
2. Please clarify why “Systemic Bias in Algorithms” is placed as one barrier in the institutional layer, rather than being treated as part of the upstream generation of the adverse decision. Conceptually, it seems different from barriers such as awareness, record access, or legal resources.
3. The strategy comparison using ANOVA needs clearer methodological explanation. Forward, backward, and greedy appear to be fixed trajectories, whereas random ordering is averaged over 10 runs. It is not clear what the ANOVA observations are, what independence assumptions are being used, or whether that comparison is statistically meaningful as presented.
4. The wording around Sobol results should be softened. “ST exceeded S1 for all barriers” is supported; “substantially exceeded” for all barriers is not.
5. The manuscript would benefit from a short subsection that distinguishes clearly between:
    - a conceptual barrier framework,
    - a formal mathematical model,
    - and claims intended to generalise to real healthcare systems.
6. Figure legibility could be improved, especially for the multi-panel sensitivity and robustness figures. The figure concepts are useful, but labels are quite dense in the PDF. The charts on pages 12–13 and the supplementary plots are informative, yet they would benefit from larger text and clearer captions that state the practical interpretation rather than only the method.

7. The supplement is concise and useful, but I would strongly encourage the author to add one more table showing the full step-by-step derivation of each pass probability from the cited source statistic. At present the supplement lists the final values, ranges, and short logic, but not the complete mapping rationale.
8. The versions of Anthropic Claude and OpenAI ChatGPT used for literature support and language refinement should be specified.

Recommendations

Recommendation: Major revision

I would encourage the editor to consider the manuscript further only if the author addresses the following core points:

- Reframe the paper more precisely as a formal or conceptual modelling study unless substantially more healthcare-specific empirical evidence is added.
- Reduce the strength of healthcare-specific and policy-effectiveness claims.
- Justify the serial multiplicative independence assumption much more rigorously, or compare it with plausible alternative recourse structures.
- Distinguish clearly between mathematical consequences of the model and empirical observations about real systems.
- Expand the derivation audit trail within the manuscript package itself.
- Moderate the robustness and interaction-language where the supplementary results do not support strong phrasing.

In its present form, the paper is thoughtful and potentially valuable, but it is not yet sufficiently grounded for the strength of its digital health claims. With substantial revision, it could become a worthwhile methodological contribution on algorithmic recourse and fairness.
