# Supplementary material for: Structural limits of single-barrier reform in algorithmic recourse: A formal series-system model with implications for digital health
Source: PLOS Digit Health. 2026 Oct 26;5(10):e0001784. doi: 10.1371/journal.pdig.0001784 (PMC13705585; doi:10.1371/journal.pdig.0001784)
Supplement: Supplementary file 3 [file pdig.0001784.s003.docx]

March 11, 2026

Dear Editors,

**Re:** Response to Resubmission Inquiry — PDIG-D-26-00342, “Synergistic barriers to algorithmic recourse in healthcare and administrative systems”

Thank you for the opportunity to clarify the submission history of this manuscript. I am writing in response to your request for a letter explaining the relationship between the current submission (PDIG-D-26-00342) and a prior submission to PLOS ONE (PONE-D-26-08047). I am happy to provide a full and accurate account.

**Submission History**

The full sequence of events is as follows:

1. An earlier version of this work, titled “Algorithmic discrimination as a synergistic barrier system: a quantitative interaction-dominant model,” was submitted to PLOS ONE (PONE-D-26-08047) and received a desk rejection without external peer review. The PLOS ONE decision letter (Dr. Daniel Parkes, Staff Editor) indicated that the submission did not appear to be motivated by a clearly outlined research question, that the mathematical insight provided was unclear, that the analysis appeared largely descriptive, and that supporting citations were insufficient. No reviewer comments were provided.

2. The manuscript was substantially revised in response to these concerns (see Revisions 1–4 below) and the title was updated to its current form. The revised version was posted to medRxiv (MEDRXIV/2026/346836) as a preprint.

3. The medRxiv preprint was transferred directly to PLOS Digital Health via the medRxiv journal transfer mechanism, on the basis that the revised manuscript’s focus on algorithmic systems in healthcare and administrative settings is a better disciplinary fit for PLOS Digital Health than for PLOS ONE.

**Revisions Made in Response to PLOS ONE Feedback**

The following substantive revisions were made between the PLOS ONE submission and the medRxiv preprint that was transferred to PLOS Digital Health:

**1. Research question sharpened and reframed toward digital health.** The revised manuscript opens with an explicit research question: under what conditions do single-barrier reforms have negligible absolute impact on algorithmic recourse outcomes, and how much of achievable improvement is attributable to cross-layer interactions? The framing was reoriented to center healthcare AI as the primary context, with credit, employment, and housing domains used as supporting analogs where empirical barrier data are available.

**2. Mathematical contribution made explicit.** Two closed-form propositions were added: Proposition 1 establishes an algebraic bound on single-barrier intervention effects as a function of baseline success probability; Proposition 2 demonstrates that interaction dominance is a structural property of multiplicative systems on the probability scale, not a simulation artefact. This directly addresses the concern that the mathematical insight was unclear.

**3. Citation base substantially expanded.** The prior version lacked sufficient grounding in the empirical and theoretical literature. The current manuscript includes 23 references spanning reliability engineering, patient safety, clinical AI fairness, algorithmic audit studies, federal datasets, and cooperative game theory, with each barrier probability traceable to a primary empirical source.

**4. Methodological transparency strengthened.** Parameter derivation traceability was substantially improved, with explicit documentation of the mapping from federal dataset statistics (CFPB, FTC, LSC) to each model parameter, conservative mapping decisions disclosed, and full derivation logic available in the public code repository and Supporting Information.

**Further Revisions Made After Transfer**

Following transfer to PLOS Digital Health, three additional revisions were made upon self-review to proactively address methodological transparency concerns. The revised manuscript submitted herewith replaces the transferred version. No analytical results, figures, or tables were changed:

**5. Circularity of validation disclosed.** A sentence was added to the limitations paragraph explicitly noting that the simulation results are consistent with but not independent of the analytical propositions, as both derive from the same multiplicative model structure. The propositions are algebraic properties of the model; the simulation provides numerical illustration rather than independent empirical validation.

**6. Sensitivity analysis scope clarified.** A sentence was added to the limitations paragraph clarifying that the reported robustness applies within the empirically bounded perturbation ranges used (±10%–33%), which were set conservatively by the author, and that true parameter uncertainty may exceed these bounds.

**7. Orphaned references integrated.** References [22] (Ajunwa, 2020) and [23] (Raghavan et al., 2020) appeared in the reference list of the transferred version but were not cited in the manuscript body. Both have been integrated into the Discussion at the sentence addressing limitations of algorithmic-layer-only fairness interventions. No new references were added.

**Rationale for PLOS Digital Health**

PLOS Digital Health is the appropriate venue for three reasons. First, the manuscript’s core contribution—demonstrating that synergistic barrier dynamics render single-target algorithmic interventions mathematically insufficient—is directly relevant to the emerging field of algorithmic health equity and digital health governance. Second, the computational methodology (multiplicative cascade models, Shapley decomposition, Sobol global sensitivity analysis) is consistent with quantitative methods published in PLOS Digital Health. Third, the finding that the dominant three-way interaction across data integration, data accuracy, and institutional access layers accounts for 87.6% of total effect variance has direct implications for how digital health systems should be audited and reformed—a question central to this journal’s mission.

**Preprint Disclosure**

The revised manuscript was posted to medRxiv (MEDRXIV/2026/346836) following the PLOS ONE desk rejection and prior to transfer to PLOS Digital Health. The medRxiv preprint remains live and has been disclosed in the submission system. The manuscript is not under consideration elsewhere. The earlier version submitted to PLOS ONE was not posted to any preprint server.

I hope this provides a complete and accurate account of the submission history and the improvements made at each stage. I welcome any further questions from the editorial office and look forward to the opportunity for peer review at PLOS Digital Health.

Sincerely,

**A.C. Demidont, DO**

Independent Researcher | Founder & CEO, Nyx Dynamics LLC

Philadelphia, Pennsylvania, USA

acdemidont@nyxdynamics.org

ORCID: 0000-0002-9216-8569
