# Supplementary material for: Structural limits of single-barrier reform in algorithmic recourse: A formal series-system model with implications for digital health
Source: PLOS Digit Health. 2026 Oct 26;5(10):e0001784. doi: 10.1371/journal.pdig.0001784 (PMC13705585; doi:10.1371/journal.pdig.0001784)
Supplement: Supplementary file 4 [file pdig.0001784.s004.docx]

# Response to Reviewers — PDIG-D-26-00342

**“Synergistic barriers to algorithmic recourse in healthcare and administrative systems”** (revised title below)

Dear Dr. Kuo and Dr. Celi,

Thank you for the opportunity to revise this manuscript, and please pass our thanks to Reviewer #1 for an unusually constructive and technically precise review. The reviewer’s central observation — that the manuscript’s strongest conclusions are properties of a formal model rather than empirical measurements of healthcare recourse, and that the healthcare framing outran the (largely cross-domain) evidence base — is correct, and we have taken the reviewer’s own recommended path: **we reposition the paper explicitly as a formal, cross-domain series-system model of algorithmic recourse with implications for digital-health governance, rather than as a healthcare-centered empirical study.**

Three structural changes organize the revision:

1. **A four-level taxonomy of claims** — (1) theorem-level results, (2) numerical/model illustrations, (3) empirical calibration assumptions, (4) real-world hypotheses — is introduced early and applied consistently. Every headline number (0.0018% baseline, 0.0054% maximum single-barrier gain, 87.6% three-way share) is now explicitly labeled as a level 1–2 property of the model, not an empirical finding about healthcare.
2. **A new quantitative robustness analysis** relaxing the multiplicative-independence assumption (Major 4), which both delimits the model’s domain of validity and, we believe, materially strengthens the paper.
3. **A full parameter-derivation audit trail** brought into the manuscript package (Major 7), plus correction of several internal inconsistencies we found during revision (detailed below), and moderation of every overstated claim (Majors 5–6).

We also disclose, in the interest of transparency, three internal discrepancies we identified and corrected while preparing this revision (they were not raised by the reviewer): a stale main-text interaction table, a rounding error in the maximum single-barrier effect, and an inaccurate characterization of the Obermeyer (2019) statistic. Details under Majors 3, 5, and 7.

The reframed working title is: **“Structural limits of single-barrier reform in algorithmic recourse: a formal series-system model with implications for digital health.”**

### Submission history (for continuity)

Further to our earlier correspondence with the editorial office: an earlier version of this work was submitted to PLOS ONE (PONE-D-26-08047) and received a **desk rejection without external peer review** (Dr. Daniel Parkes, Staff Editor), on grounds of presentation and scope rather than scientific validity. The manuscript was then substantially revised — the research question was sharpened, two closed-form propositions were added, and the reference base and parameter-derivation traceability were expanded — posted to medRxiv (doi:10.64898/2026.02.22.26346836), and transferred to PLOS Digital Health via the medRxiv journal-transfer mechanism. The manuscript is not under consideration elsewhere, and the preprint is disclosed in the submission system. The present revision reframes the paper further in direct response to Reviewer #1 below. (Where our earlier transfer letter described Proposition 2 as establishing “interaction dominance,” we have since corrected that characterization — see Major 4 — because dominance is parameterization-dependent, not a structural property.)

## Editor requirements (addressed in the submission form)

- **CRediT:** Full CRediT contributions for the sole author (A.C. Demidont) completed in the submission form (Conceptualization, Methodology, Software, Formal analysis, Investigation, Writing – original draft, Writing – review & editing).
- **Competing interests:** Provided in required PLOS format in the form (prior Gilead employment/stock fully divested Dec 2024, no role in this work; ownership of Nyx Dynamics LLC; research conducted independently).
- **Figures:** All figures regenerated at PLOS-compliant resolution and provided as separate **.tif** files (validated via the PLOS NAAS tool).

## Reviewer #1

We are grateful for the reviewer’s assessment that the work is “ambitious and intellectually interesting,” the research question “explicit,” the propositions “easy to follow,” and the limitations disclosure “commendabl[e].” We have retained those strengths while addressing each concern.

### Major 1 — Headline findings are consequences of model structure, not independent empirical discoveries

**Comment (quoted):** “the strongest conclusions are driven primarily by the model structure rather than by healthcare-specific empirical evidence … the reader should not interpret the bootstrap, Sobol, Morris, and strategy-removal analyses as independent empirical confirmation … The paper would be much stronger if it separated: theorem-level results … simulation-based illustrations … empirical assumptions … and real-world claims.”

**Response:** We agree entirely. This is now the organizing principle of the paper.

**Action taken:** - Added a Methods subsection, **“Scope of claims: conceptual framework, formal model, empirical calibration, and real-system hypotheses”**, defining the four-level taxonomy and stating explicitly which results are which. - Relabeled every headline quantity as a **model-derived** result (level 1–2). The abstract, Results, and Discussion now use “illustrated by,” “consistent with,” and “implies under the model,” and never “confirmed/validated by” for the Shapley/Sobol/Morris/bootstrap analyses. - Added a sentence clarifying that the simulation outputs derive from the same multiplicative structure as the propositions and therefore illustrate, rather than independently confirm, them.

### Major 2 — Healthcare framing not sufficiently supported by the parameter sources

**Comment (quoted):** “the barrier table shows that many model inputs are derived from credit reporting, dispute resolution, legal access, and employment-related sources … the majority of parameter values are not measured in healthcare recourse pathways … narrow the claims and title so that the paper is explicitly cross-domain.”

**Response:** Agreed; we have taken option (3), narrowing the claims and title. Ten of eleven parameters are drawn from cross-domain (CFPB/FTC/LSC) sources, and we no longer assert healthcare as the primary empirical context.

**Action taken:** - New title (above) foregrounds the formal model and “cross-domain … with implications for digital health,” and drops the healthcare empirical assertion. - Abstract and Introduction reframed: healthcare is now a **motivating application** and the target of digital-health **implications**, with credit/employment/housing as the co-equal domains from which parameters are actually drawn. - The transportability limitation is stated explicitly in both the derivation table (Major 7) and the Discussion.

### Major 3 — Over-reliance on Obermeyer 2019; needs scope/limits and post-2019 context

**Comment (quoted):** “relies heavily on Obermeyer et al. 2019 … does not sufficiently discuss the scope and limits of that study … cannot on its own support a comprehensive account of healthcare algorithmic bias.”

**Response:** Agreed. We add a scope/limits paragraph and contextualize with current literature, and we demote Obermeyer from an empirical anchor to one motivating example. **Separately, we discovered and corrected an accuracy error:** the prior text described Obermeyer as showing “approximately 62% undertriage of Black patients.” That figure is not supported by Obermeyer et al.; their reported result is that correcting the cost-for-need proxy would raise the fraction of Black patients receiving additional help from 17.7% to 46.5% (with ~26.3% more chronic conditions at equal risk score). The text and the Systemic-Bias parameter rationale have been corrected accordingly.

**Action taken:** - Added an Obermeyer scope/limits paragraph (single commercial risk tool, single health system, cost-as-need proxy mechanism; context-specific, not a population-wide calibration). - Added a “field has evolved since 2019” paragraph citing Seyyed-Kalantari et al. (2021, *Nat Med*, underdiagnosis bias in chest radiographs), Gichoya et al. (2022, *Lancet Digit Health*, model recovery of race from images), and Arora/Alderman et al. (2023, *Nat Med*, dataset-diversity standards), alongside the already-cited clinical-fairness and STANDING Together works. - Corrected the Obermeyer statistic throughout.

### Major 4 — Serial multiplicative independence is under-justified and likely drives the result

**Comment (quoted):** “interaction dominance is almost built into the series formulation … justify much more rigorously why the serial multiplicative topology is the appropriate abstraction, or include at least one comparison with a plausible alternative structure.”

**Response:** This was the most important comment, and we have addressed it with new computation rather than prose. Two clarifications and one new analysis:

1. *Serial ≠ independent.* We now separate (A) the **serial/all-required topology** from (B) the **calibration** of stage probabilities. A sequential product is simply the chain rule, P(S₁…Sₙ)=P(S₁)∏P(Sᵢ|S₁…Sᵢ₋₁), and does **not** require independence. The genuine vulnerability the reviewer identifies is that the implemented pᵢ are largely *marginal/proxy* statistics standing in for *conditional* transition probabilities. We now state this explicitly.
2. *New robustness analysis (added to Methods/Results and the repository as copula_robustness.py, seed 42).* We relaxed independence with a **Gaussian copula** over the 11 Bernoulli pass-events (equicorrelation ρ = 0→0.5, and a within-layer “block” variant, n = 100,000), and separately tested a **repeated-attempt topology** (pᵢ^(m)=1−(1−pᵢ)^m, m = 1,2,3).

**Findings (now reported in full):** - The independence case reproduces the submitted values exactly (baseline 0.0018%, three-way share 87.6%). - Under correlation up to ρ = 0.5, **interaction dominance is robust** (three-way share stays 77–88%) and **single-lever reform remains far weaker than coordinated reform** (at ρ = 0.5, best single-layer gain 4.5% vs 96.8% for coordinated removal). However, the **baseline point value is correlation-sensitive**, rising from 0.0018% toward the weakest barrier (3.2% at ρ = 0.5) — we now concede this explicitly. - Under **repeated attempts**, the strong conclusion **weakens** (three-way share falls to 53% at m = 2 and 25% at m = 3), which honestly **delimits the model’s domain of validity** to approximately single-pass, serial recourse.

**Action taken:** New Methods subsection “Why a serial multiplicative topology, and what changes if it is wrong”; new Results subsection and figure reporting the ρ- and m-sweeps; Discussion now states which conclusions are structural (the comparative/threshold shape, robust to correlation) and which are topology-dependent (the specific baseline value and 87.6% point estimate). Per the reviewer, attenuation under alternative topologies is presented as informative domain-of-validity evidence, not failure.

### Major 5 — Interpretive claims stronger than the supporting results

**Comment (quoted):** “‘substantially exceeding first-order indices for all barriers’ … the ST–S1 gap is very small for some barriers, especially Awareness Gap (0.112 vs 0.109) and Legal Knowledge Gap (0.111 vs 0.110) … the overall presentation still leans too heavily on the language of robustness.”

**Response:** Agreed on both counts.

**Action taken:** - We now seed all stochastic analyses (see Major 7); this makes the Sobol/CV values reproducible but shifts them slightly from the unseeded values the reviewer cited. We report this transparently rather than silently. - Sobol wording changed to: “total-order indices exceeded first-order indices for all barriers, but the gaps are small and, for several barriers, within Monte-Carlo error at the current sample size—so ‘substantially exceeded for all barriers’ is not supported.” (The reviewer correctly cited very small gaps for Awareness Gap and Legal Knowledge Gap in the unseeded run; under the seeded canonical run the smallest gaps fall on Correction Process/Legal Resource, which only reinforces that these per-barrier orderings are sampling-sensitive.) - Robustness language replaced throughout with bounded-perturbation phrasing (“stable within the ±10–33% perturbation ranges”; “100% robustness” removed). On the coefficient of variation: the previously reported unseeded values (73.2% at 25%, 93.8% at 30%) are replaced by seeded canonical values that are **even less robust** (≈110% at 25%, ≈129% at 30%; CV crosses 100% by 25% noise). Robustness is explicitly **not** claimed under realistic uncertainty. The per-barrier Sobol/Shapley orderings that shift under re-seeding do so within Monte-Carlo error and are reported with that caveat; the deterministic headline values (baseline, maximum single-barrier effect, three-way share) are unaffected. - *Discovered/corrected inconsistency:* the main-text ANOVA table reported pairwise interactions (L1×L2 = 3.4%, L1×L3 = 8.0%, L2×L3 = 0.5%) that disagreed with the SI table, the Fig 3 heatmap, and the code. The correct values (matching all three) are L1×L2 = 0.44%, L1×L3 = 4.51%, **L2×L3 = 7.03%** (Data Accuracy × Institutional is the largest pairwise term). The main-text table has been corrected.

### Major 6 — Policy implications overstated relative to the evidence base

**Comment (quoted):** “It cannot yet firmly conclude that current healthcare audit or notification policies are ineffective in practice. Those statements should be reframed as implications or hypotheses.”

**Response:** Agreed.

**Action taken:** All policy statements recast conditionally, e.g., “**if** recourse behaves as a low-baseline serial barrier system, single-layer interventions are **unlikely** to produce large absolute gains — a testable hypothesis, not a demonstrated policy fact.” The claim that reforms “have had limited impact” because of the model is removed; the model now yields hypotheses for digital-health governance, explicitly pending validation against observed recourse trajectories.

### Major 7 — Derivation audit trail incomplete within the submitted materials

**Comment (quoted):** “the steps from source statistics to each pass probability should be reproducible from the manuscript package alone.”

**Response:** Agreed. We have built a complete derivation audit trail and brought it into the package. (We also note the referenced repository file data/parameter_sources/parameter_derivation.md was missing in the submitted version; it has now been created.)

**Action taken:** - New **Supplementary Table S6**: one row per barrier giving the source domain, the exact source statistic and citation, the resulting pass probability pᵢ, its plausibility range, and an explicit transportability limitation — making each parameter auditable from the manuscript package. The target construct and full step-by-step mapping rule for every parameter are provided in the repository derivation file (data/parameter_sources/parameter_derivation.md). - The same content is mirrored in the repository as data/parameter_sources/parameter_derivation.md, generated by a committed script (build_derivation_table.py) so the paper and code cannot drift.

### Minor comments

- **Minor 1 (define “successful recourse”):** Defined operationally as **full end-to-end resolution** (underlying data corrected **and** adverse decision reversed **and** correction propagated across institutions); we note this stringent endpoint is itself a modeling choice that lowers the baseline, and we flag less-stringent endpoints as alternatives.
- **Minor 2 (Systemic Bias placement):** Rather than argue placement rhetorically, we **ran the sensitivity test** (systemic_bias_sensitivity.py, seed 42): a 10-barrier model with “Systemic Bias in Algorithms” removed/reclassified as upstream. The qualitative conclusions are unchanged — baseline rises from 0.0018% to 0.0060% (still ≪1%), the three-way interaction share stays dominant (86.6% vs 87.6%), and the maximum single-barrier effect stays <0.02% (0.0180%). We retain the barrier in the cascade, note its most natural reading is bias *persistence during recourse* (not generation of the initial decision), and state explicitly that the results do not depend on this choice.
- **Minor 3 (strategy ANOVA):** The inferential ANOVA (F = 0.07, p = 0.98) compared three deterministic trajectories against one averaged random trajectory and lacked a defensible sampling model. We have **removed the F-test** and retained a purely descriptive statement that all strategies converge only at near-complete removal.
- **Minor 4 (Sobol wording):** Addressed with Major 5.
- **Minor 5 (scope subsection):** Added (see Major 1).
- **Minor 6 (figure legibility):** All multipanel figures regenerated with larger fonts and cleaner layout; captions rewritten to state the practical interpretation, not only the method; provided as .tif.
- **Minor 7 (derivation table):** Added (see Major 7).
- **Minor 8 (AI tool versions):** The AI-use statement now names each tool and its role (Anthropic Claude and OpenAI ChatGPT for literature support and language refinement; JetBrains Junie for code correction; Zotero for reference management). Specific model versions/dates were not systematically recorded at the time of use; we state this explicitly and do not assert unverified version strings, consistent with transparent disclosure.

## Summary of new analyses and artifacts in this revision

- Correlated-barrier (Gaussian copula) and repeated-attempt robustness analyses (copula_robustness.py), seeded and reproducible.
- Systemic-Bias 10-barrier sensitivity test.
- Full parameter-derivation table (supplement + repository).
- Seeded, deterministic reproduction of all stochastic results (Shapley/Sobol/Morris/bootstrap), with a one-command make pipeline and regression tests; the previous submission’s stochastic values came from an unseeded run and are now replaced by seeded canonical values.
- Corrections to three internal inconsistencies (interaction table; 0.0055%→0.0054%; Obermeyer statistic).

We believe the revised manuscript is a more honest and more useful contribution — a clearly delimited formal model whose scope and robustness are now explicit — and we thank the reviewer again for guidance that materially improved it.

Sincerely, A.C. Demidont, DO
